# Temporal processing and long-latency auditory evoked potential in stutterers^☆^

**DOI:** 10.1016/j.bjorl.2016.02.015

**Published:** 2016-04-28

**Authors:** Raquel Prestes, Adriana Neves de Andrade, Renata Beatriz Fernandes Santos, Andrea Tortosa Marangoni, Ana Maria Schiefer, Daniela Gil

**Affiliations:** Universidade Federal de São Paulo (UNIFESP), Departamento de Fonoaudiologia, São Paulo, SP, Brazil

**Keywords:** Stuttering adult, Auditory processing disorder, Auditory evoked potential, Gagueira adulta, Distúrbio do processamento auditivo, Potencial evocado auditivo

## Abstract

**Introduction:**

Stuttering is a speech fluency disorder, and may be associated with neuroaudiological factors linked to central auditory processing, including changes in auditory processing skills and temporal resolution.

**Objective:**

To characterize the temporal processing and long-latency auditory evoked potential in stutterers and to compare them with non-stutterers.

**Methods:**

The study included 41 right-handed subjects, aged 18–46 years, divided into two groups: stutterers (*n* = 20) and non-stutters (*n* = 21), compared according to age, education, and sex. All subjects were submitted to the duration pattern tests, random gap detection test, and long-latency auditory evoked potential.

**Results:**

Individuals who stutter showed poorer performance on Duration Pattern and Random Gap Detection tests when compared with fluent individuals. In the long-latency auditory evoked potential, there was a difference in the latency of N2 and P3 components; stutterers had higher latency values.

**Conclusion:**

Stutterers have poor performance in temporal processing and higher latency values for N2 and P3 components.

## Resumo

**Introdução:**

A gagueira é um distúrbio da fluência da fala, e pode estar associada a fatores neuroaudiológicos ligados ao processamento auditivo central, entre eles as alterações das habilidades auditivas de processamento e resolução temporal.

**Objetivo:**

Caracterizar o processamento temporal e o potencial evocado auditivo de longa latência em indivíduos gagos, e compará-los com indivíduos sem gagueira.

**Método:**

Participaram do estudo 41 indivíduos destros, na faixa de 18 a 46 anos de idade, distribuídos em dois grupos: 20 com gagueira e 21 sem gagueira, compararados segundo idade, escolaridade e gênero. Todos os indivíduos foram submetidos aos testes de padrão de duração, teste de identificação de intervalos aleatórios e o potencial evocado auditivo de longa latência.

**Resultados:**

Indivíduos com gagueira apresentaram pior desempenho nos testes de padrão de duração e *Random Gap Detection*, quando comparados aos indivíduos fluentes. No potencial evocado auditivo de longa latência, houve diferença na latência dos componentes N2 e P3, sendo que os indivíduos gagos apresentaram maiores valores de latência.

**Conclusão:**

Os indivíduos com gagueira apresentaram processamento temporal com desempenho abaixo do esperado e um maior valor de latência para os componentes N2 e P3.

## Introduction

Individuals with speech and language disorders may exhibit changes in the processing of information received through the sense of hearing. Studies have shown that there is a correlation between the processing of auditory information, visual pathways, and expressive language difficulty, which can affect speech fluency and be characterized as stuttering.^1^, ^2^, ^3^

Stuttering is known as a break in the flow of speech, a multifactorial disorder in which biological, psychological, and social aspects are correlated in a complex manner.^1^ Changes in perception or auditory information processing are among the biological aspects.^2^, ^4^, ^5^, ^6^

The processing of auditory information is related to the temporality of the sounds, rhythm, and prosody, aspects in which stutterers may show changes.^1^ Particularly when the degree of dysfluency is severe, the processing abnormalities in these areas have been proposed as the immediate cause of stuttering, since temporal auditory processing is critical for speech perception and closely related to the spoken language processing.^1^, ^7^, ^8^ Therefore, it is necessary to evaluate the neuroaudiological processes of this population, which can be performed through auditory behavioral tests and auditory evoked potentials.

Auditory temporal processing refers to an individual's ability to detect changes in temporal features of sounds, such as duration, intensity, frequency, and pauses between stimuli.^9^ There are several procedures available to evaluate the temporal processing aurally, such as the tests of frequency pattern and duration with pure tone^10^, ^11^, ^12^ and musical tone.^13^ The discrimination of pauses between stimuli can be evaluated with the with the Random Gap Detection Test (RGDT).^14^

In a study of children with developmental stuttering that evaluated the temporal patterns (frequency and duration aspects), it was found that stuttering children had worse performance and greater number of changes compared to their non-stuttering peers.^1^ Some authors have studied the temporal resolution and observed worse performance in stutterers.^15^, ^16^

One of the electrophysiological procedures available to evaluate the aspects related to attention, memory, and auditory discrimination^17^ is the long-latency auditory evoked potential (LLAEP). Studies that investigated stuttering and the LLAEP have reported differences in P3 amplitudes, with stutterers showing a lower amplitude.^4^, ^18^ In another study, there was no difference in latencies and amplitudes of P3 between stutterers and non-stutterers.^19^

Recent national studies showed no differences in P300 latencies when comparing stutterers and non-stutterers.^5^, ^6^

Given these findings in the literature, it is evident that there are links between the auditory ability of temporal processing and the occurrence of stuttering. However, there is still no consensus on the way through which the skills of temporal processing, long-latency auditory evoked potentials, and manifestation of stuttering correlate. One hypothesis for the lack of consensus in the literature would be the heterogeneity of stutterers groups in each study. Moreover, as stuttering is a multifactorial disorder with biological, psychosocial, and environmental influences, such factors can also lead to inconclusive results, requiring more studies to increase the knowledge about these relationships.

Thus, the aim of this study was to characterize temporal processing and long-latency auditory evoked potential in stutterers and compare with non-stutterers.

## Methods

The study design was approved by the Research Ethics Committee under the No. 26574/2012. A cross-sectional and observational study was performed; the sample of stutterers was selected at an evaluation and diagnostic audiology service from a school hospital, and the sample of non-stutterers, consisting of volunteers, was selected by convenience, matching the stutterers regarding age, education, and sex.

For participation in this study, the following inclusion criteria were established: right-hand preference, ages between 18 and 55 years, auditory thresholds within normal limits (with thresholds of up to 25 dB HL in sound frequencies of 250–258 kHz), type-A tympanometric curve and presence of contralateral stapedial acoustic reflex (in sound frequencies of 500–504 kHz), and negative history of conductive hearing loss and/or neurological disorders.

Also, for the group of stutterers (GS), subjects had mild to moderate stuttering, according to the Stuttering Severity Instrument (SSI-3),^20^ and for the comparison group of non-stutterers (GNS), subjects should have no speech dysfluencies.

Behavioral assessment of temporal processing was performed with the aid of the following equipment: soundproof booth, Philips Expanium discman, Grason-Stadler GSI-61 audiometer and supra-aural TDH-50P earphones, CompactDisc with Duration Pattern Test (DPT)^11^ and RGDT.^14^

The DPT with pure tone^11^ was presented binaurally at 50 dB HL, based on the average of the auditory thresholds of 500 Hz, 1000 Hz, and 2000 Hz. Thirty sequences were presented binaurally, with six possible combinations (LLC, CCL, LCL, CLC, LCC, and CLL). Subjects were asked to name the combination presented to them. The normality criterion used was at least 83% of correct answers.^10^, ^11^

RGDT was presented binaurally at 50 dB HL, based on the average of the auditory thresholds of 500 Hz, 1000 Hz, and 2000 Hz. The test began with the presentation of a training range, with 0.5 kHz stimuli in which the inter-stimulus intervals varied from zero to 40 ms, appearing in an increasing manner, that is: 0; 2; 5; 10; 15; 20; 25; 30; and 40 ms. After successful training, subtests were initiated in frequencies of 0.5 k; 1 k; 2 k; and 4 kHz. In each frequency, sequences of nine stimuli were presented, with randomly distributed gaps.^14^ For each stimulus, the subject was instructed to report if he/she had detected one or two tones; that is, identified the presence of a gap (interval). The normality criterion was the average gap below 10 ms.^21^

For electrophysiological evaluation, the two-channel equipment (Smart EP USB Jr Intelligent Hearing Systems [his]), insert earphones (ER-3A), abrasive paste, electrolytic paste, microporous tape, and silver electrodes were used. The auditory evoked potentials were obtained in an acoustically and electrically treated room, with the subject accommodated comfortably in a reclining chair. After cleaning the skin with abrasive paste, the electrodes were fixed with adhesive tape and arranged in the 10–20 system^22^ on the vertex (Cz), earlobes (A1 left and A2 right), and on the forehead (the ground, Fpz). The impedance of the electrodes was then checked, so that the values were equal to or less than 5 kΩ.^23^

To capture the LLAEP, the tone-burst stimuli was used, presented binaurally at 70 dB HL, in the frequencies of 1000 Hz (frequent stimulus that represented 80% of the stimuli) and 2000 Hz (rare stimulus that represented 20% of the stimuli), with a total of 300 stimuli, with 1.1 second presentation rate, with alternating polarity, filters at 1–30 Hz, and a 600 ms window. N1, N2, and P2 components were analyzed in the trace of frequent stimulus, while P3 was analyzed in the trace resulting from the subtraction of frequent stimulus by the rare stimulus. The normal range for each component is^24^: N1, 80–150 ms; P2, 145–180 ms; N2, 180–250 ms; 17–20 years of age, 225–365 ms; and 30–50 years of age, 290–380 ms.

For statistical analysis, Mann–Whitney tests (for DPT), Friedman test (for RGDT), and nonparametric analysis of variance with repeated measures (for LLAEP) were used to determine whether there were differences between the groups (GS and GNS). A significance level of 5% was adopted, and tests with statistically significant results were highlighted with an asterisk (*).

## Results

Forty-one subjects, aged 18–46 years, of both sexes, were divided into two groups: group of stutterers (GS), consisting of 20 subjects with mild to moderate stuttering, nine females and 11 males, and group of non-stutterers (GNS), consisting of 21 non-stuttering individuals, ten females and 11 males. The mean age for both groups was 30 years.

The Mann–Whitney test was used for DPT and the Friedman test, for RGDT.

The statistical analysis technique used was nonparametric analysis of variance with repeated measures.

In the assessment of temporal processing (Table 1), statistically significant differences were found in the results of the DPT and RGDT between the GS and the GNS, with the GS presenting the lower percentage of correct answers in DPT and higher values in gap detection compared to the GNS, the values of both DPT and RGDT were abnormal in the GS and normal in the GNS.

**Table 1:** Descriptive measures of the DPT (%) and RGDT (ms) responses in the GNS and GS groups.

Variable	Group	*N*	Mean	Standard deviation	*p*-Value
DPT (%)	GNS	21	89.2	7.9	0.009^a^
	GS	20	74.8	21.5	
RGDT (ms)	GNS	21	7.3	2.7	0.012^a^
	GS	20	10.4	7.1	

DPT, Duration Pattern Test; RGDT, Random Gap Detection Test; *N*, sample size; GNS, group of non-stutterers; GS, group of stutterers.

a Statistically significant.

In the evaluation of LLAEP (Table 2), there were no differences between the latency values in both groups for N1 and P2 components. A difference was observed only between the right and left ears for both groups in the N1 component, with the right ear presenting lower latency than the left ear for this component. As for the N2 and P3 components, it was found that GS latencies were greater than in GNS. There were no significant differences between the latencies of the right and left ears in both groups.

**Table 2:** Descriptive measures of the N1, P2, N2, and P3 latencies (ms) in the GNS and GS groups per ear.

Wave	Ear	Group	*N*	Mean	Standard deviation	*p*-Value between groups	*p*-Value between ears
N1	Right	GNS	21	99.7	8.8	0.376	0.006^a^
		GS	20	96.2	10.8		
	Left	GNS	21	99.9	8.7	0.376	
		GS	20	99.7	10.8		
P2	Right	GNS	21	174.1	26.0	0.902	0.650
		GS	20	167.7	25.1		
	Left	GNS	21	169.0	24.6	0.902	
		GS	20	172.0	25.1		
N2	Right	GNS	21	221.2	39.9	0.003^a^	0.526
		GS	20	245.5	48.9		
	Left	GNS	21	214.7	40.1	0.003^a^	
		GS	20	247.0	45.6		
P3	Right	GNS	21	293.7	23.0	0.006^a^	0.438
		GS	20	328.7	56.4		
	Left	GNS	21	289.0	30.4	0.006^a^	
		GS	20	332.7	61.8		

*N*, sample size; GNS, group of non-stutterers; GS, group of stutterers.

a Statistically significant.

## Discussion

The present study assessed the behavioral aspects of auditory temporal processing tests and the capture of electrophysiological potential that are related to attention, discrimination, and auditory memory, and found a discrepancy in individuals who stutter when compared with non-stutterers.

In DPT and RGDT tests, there was underperformance in GS compared to GNS (Table 1), demonstrating that individuals who stutter have difficulty discriminating sound patterns in relation to their duration, and also with respect to inter-stimulus intervals.^25^ The same findings were described by Blood, Andrade and Schochat, Andrade et al., and Schiefer and Arcuri,^26^, ^27^, ^28^, ^29^, ^30^ who compared stutterers and non-stutterers and found that stuttering is related to changes in the temporal aspects of sound processing.

The GS had statistically significantly higher RGDT thresholds when compared with the GNS. The auditory skill evaluated in this test is associated with the recognition of speech sounds, changes in duration, gaps, and rate of syllables.^25^ These aspects are important for the auditory feedback; a study with computer model that reproduced stuttering found a delayed auditory feedback in this model.^31^ There is no agreement in the literature regarding the temporal resolution of individuals who stutter. Gonçalves and Arcuri^6^, ^30^ found no differences between stutterers and non-stutterers when applying such tests in their samples, but Andrade^28^ found responses similar to the present study, showing the relationship between the temporal processing and the manifestation of stuttering in this population. The fact that there is no agreement on its association can be justified by previous studies that were conducted with a non-homogeneous sample on the degree of stuttering severity, education, and age. It is known that such factors affect the results of auditory processing tests. The temporal imprecision in speech perception, as well as the change in auditory feedback^32^ may contribute to the moments of dysfluency,^33^ explaining the low performance of GS in DPT and RGDT tests in this study.

As for LLAEP, there were significant differences in N2 and P3 latencies for both the right and left ears; the GS presented the longer latency (Table 2). Some authors found no P300 differences compared to stutterers and non-stutterers.^19^ In more recent studies, more altered results were observed in stutterers.^5^, ^6^ No studies analyzing the N2 component in individuals who stutter were retrieved. Thus, it can be hypothesized that stutterers require more time to elicit this component, affecting the speed of the auditory processing of sound response, which also explain the high rate of change in the tests evaluating the temporal processing skill applied in this study.

In the present study, although significant differences in LLAEP latency were observed, they represented no change in this potential. Unlike behavioral tests, these data can be explained by the fact that the normal range for LLAEP latency is quite wide and the components’ generating sites are diffused in the central auditory system; thus, it was observed that the auditory processing behavioral tests were more sensitive for this population.

Although the findings were relevant, this study was performed with a small sample in which the majority of stutterers had a mild degree of the condition. Therefore, more studies evaluating the temporal processing in stutterers should be performed, involving other temporal processing tests with larger and more homogeneous populations regarding the severity of stuttering, so that the findings can be generalized to the entire population of stutterers.

Despite these limitations, greater knowledge of hearing disabilities in the population of stutterers contributes to a better treatment planning and possible intervention, which may include acoustically controlled auditory training, aiming for an improvement in hearing that may lead to an improvement in speech fluency.

## Conclusion

Individuals who stutter presented poorer than expected results in the behavioral tests that assess temporal processing, as well as longer latencies in long-latency auditory evoked potential.

## Conflicts of interest

The authors declare no conflicts of interest.

## References

[1] Silva R., Oliveira C.M.C., Cardoso A.C.V. (2011). Aplicação dos testes de padrão temporal em crianças com gagueira desenvolvimental persistente. Rev CEFAC.

[2] Jutras B., Lagacé J., Lavigne A., Boissonneault A., Lavoie C. (2007). Auditory processing disorders, verbal disfluency, and learning difficulties: a case study. Int J Audiol.

[3] Oliveira A.M.C.C., Ribeiro I.M., Merlo S., Chiappetta A.L.M.L. (2007). O que fonoaudiólogos e estudantes de fonoaudiologia entendem por fluência e disfluência. Rev CEFAC.

[4] Hampton A., Weber-Fox C. (2008). Non-linguistic auditory processing in stuttering: evidence from behavior and event-related potencials. J Fluency Disord.

[5] Angrisani R.M.G., Matas C.G., Neves I.F., Sassi F.C., Andrade C.R.F. (2009). Avaliação eletrofisiológica da audição em gagos, pré e pós terapia fonoaudiológica. Pró-Fono R Atual Cient.

[6] Gonçalves I.C. (2013).

[7] Foundas A.L., Corey D.M., Hurley M.M., Heilman K.M. (2004). Verbal dichotic listening in developmental stuttering: subgroups with atypical auditory processing. Cogn Behav Neurol.

[8] Guitar B. (2006).

[9] Musiek F., Shinn J., Jirsa R., Bamiou D., Baran J., Zaidan E. (2005). The GIN (Gaps in Noise) test performance in subjects with and without confirmed central auditory nervous system involvement. Ear Hear.

[10] Corazza M.C.A. (1998).

[11] Musiek F.E. (1994). Frequency (pitch) and duration patterns testes. J Am Acad Audiol.

[12] (1997). Auditec Evaluation manual of pitch pattern sequence and duration pattern sequence. Saint Louis.

[13] Taborga MBL. Processos temporais auditivos em músicos de Petropólis. Rio de Janeiro. Universidade Pontifícia de Petropólis e Universidade Federal de São Paulo, 1999 monografia.

[14] Keith R.W. (2000).

[15] Liebetrau R.M., Daly D.A. (1981). Auditory processing perceptual abilities of organic and functional stutterers. J Fluency Disord.

[16] Kramer M.B., Green D., Guitar B. (1987). A comparison of stutters and nonstutters on masking level differences and synthetic sentence identification tasks. J Com Disord.

[17] Schochat E. Potenciais Evocados Auditivos. In: Carvallo RMM. Fonoaudiologia Informação para formação: Procedimentos em audiologia pp. 57-70; 2003.

[18] Morgan M.D., Cranford J.L., Burk K. (1997). P300 event-related potentials in stutterers and nonstutterers. J Speech Lang Hear Res.

[19] Khedr M., El Nasser W.A., Abdel-Haleem E.K., Bakr M.S., Trakhan M.N. (2000). Evoked potentials and eletroencephalography in stuttering. Folia Phoniatr Logop.

[20] Riley G.D. (1994). Stuttering severity instrument for children and adults-SSI. Austin.

[21] Ziliotto K., Pereira L.D. (2005). 17th American Academy of Audiology–Annual Convention and Exposition.

[22] Jasper H.A. (1958). The ten-twenty system of the international federation. Electroencephalogr Neurol Clin Neurophysiol.

[23] Hall J. (2006).

[24] McPherson D.L. (1996).

[25] Schneider B.A., Pichora-Fuller K. (2001). Age-related changes in temporal processing: implications for speech perception. Sem Hear.

[26] Blood I.M. (1996). Disruputions in auditory and temporal processing in adults who stutter. Percept Mot Skills.

[27] Andrade C.R.F., Schochat E. (1999). Comparação entre os achados neurolinguísticos e neuroaudiológicos das gagueiras. Pró-fono.

[28] Andrade A.N., Gil D., Schiefer A.M., Pereira L.D. (2008). Avaliação comportamental do processamento auditivo em indivíduos gagos. Pró-fono.

[29] Schiefer A.M., Pereira L.D., Barbosa L.G. (1999). Considerações preliminares entre uma possível correlação entre gagueira e os aspectos linguísticos e auditivos. Pró-fono.

[30] Arcuri C.F. (2012).

[31] Cai S., Tourville J.A., Beal D.S., Perkell J.S., Guenther F.H., Ghosh S.S. (2014). Diffusion imaging of cerebral white matter in persons who stutter: evidence for network-level anomalies. Front Hum Neurosci.

[32] Oreu C., Stephen M.T., Guenther F.H. (2010). Overreliance on auditory feedback may lead to sound/syllable repetitions: simulations of stuttering and fluency-inducing conditions with a neural model of speech production. J Fluency Disord.

[33] Meyers S.C., Hughes L.F., Schoeny Z.G. (1989). Temporal-phonemic processing skills in adult stutters and no stutters. J Speech Lang Hear Res.

